# Postprandial lipemia: factoring in lipemic response for ranking foods for their healthiness

**DOI:** 10.1186/s12944-017-0568-5

**Published:** 2017-09-18

**Authors:** Cintia Botelho Dias, Paul J. Moughan, Lisa G. Wood, Harjinder Singh, Manohar L. Garg

**Affiliations:** 1 Nutraceuticals Research Program, School of Biomedical Sciences & Pharmacy, University of Newcastle, Callaghan, NSW Australia; 2 Riddet Institute, Massey University, Palmerston North, New Zealand; 3 Centre for Asthma and Respiratory Disease, School of Biomedical Sciences & Pharmacy, University of Newcastle, New Lambton, Australia; 4 Priority Research Centre in Physical Activity & Nutrition, University of Newcastle, University of Newcastle, Callaghan, NSW 2308 Australia

**Keywords:** Postprandial lipemia, Lipemic load, Triglyceridemia

## Abstract

One of the limitations for ranking foods and meals for healthiness on the basis of the glycaemic index (GI) is that the GI is subject to manipulation by addition of fat. Postprandial lipemia, defined as a rise in circulating triglyceride containing lipoproteins following consumption of a meal, has been recognised as a risk factor for the development of cardiovascular disease and other chronic diseases. Many non-modifiable factors (pathological conditions, genetic background, age, sex and menopausal status) and life-style factors (physical activity, smoking, alcohol and medication use, dietary choices) may modulate postprandial lipemia. The structure and the composition of a food or a meal consumed also plays an important role in the rate of postprandial appearance and clearance of triglycerides in the blood. However, a major difficulty in grading foods, meals and diets according to their potential to elevate postprandial triglyceride levels has been the lack of a standardised marker that takes into consideration both the general characteristics of the food and the food’s fat composition and quantity. The release rate of lipids from the food matrix during digestion also has an important role in determining the postprandial lipemic effects of a food product. This article reviews the factors that have been shown to influence postprandial lipemia with a view to develop a novel index for ranking foods according to their healthiness. This index should take into consideration not only the glycaemic but also lipemic responses.

## Background

Fasting and postprandial blood triglyceride levels are risk factors for cardiovascular and other chronic diseases [1]. Although fasting blood lipid levels indicate cumulative effects of composite diets and metabolic activity, they do not reflect accurately the impact of individual foods or meals consumed during the day. Typically, humans are in an absorptive state (non-fasting) for over 18 h in a day and therefore, postprandial triglyceride levels are now recognised as an important risk factor for cardiovascular disease [2]. Despite providing key substrates in metabolic pathways and being source of energy, fatty acids can be detrimental if in excess in the circulation. Excess fat consumption can induce a lipotoxic state, involving activation of various inflammatory pathways [3]. As early as one hour after consumption of a high fat meal, nuclear factor-kB, a key regulator of fat-induced inflammation [4, 5], is activated [6, 7], likely due to the activation of cell surface receptors by free fatty acids [8–10]. This leads to increased expression of pro-inflammatory mediators, including interleukin-6 (IL-6), tumour necrosis α (TNF-α) and interleukin-8 (IL-8) [10, 11]. In addition, oxidative stress may be triggered by an increase in the generation of reactive oxygen species by mononuclear cells and polymorphonuclear leukocytes [6, 7, 12] and an increase in other markers of oxidative stress [12, 13], one to three hours postprandially.

**Fig. 1:**
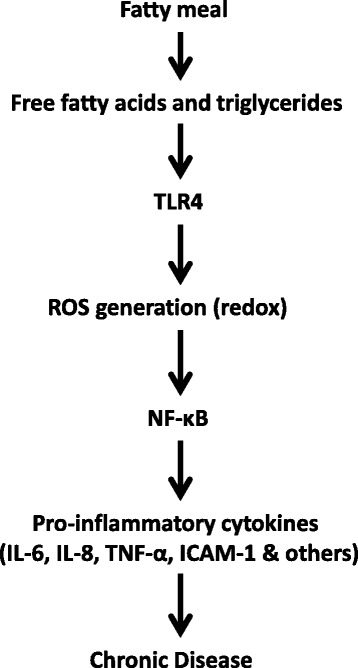
Summary of the pathophysiological effects of postprandial hypertriglyceridemia. ICAM-1, Intercellular Adhesion Molecule 1; IL-6, interleukin-6; IL-8, interleukin-8; NF-κB, nuclear factor κB; ROS, reactive oxygen species; TLR4, toll like receptor 4; TNF-α, tumour necrosis factor-α

Indeed, the oxidative degradation of fatty acids and the transient production of pro-inflammatory mediators, as nutrients are metabolised, are appropriate homeostatic responses. However, these responses become undesirable when the host is unable to efficiently clear nutrients that are consumed in excess. The response to metabolic surplus can include various adverse outcomes, such as vascular events [14, 15], insulin resistance [16] or inflammatory cell recruitment [17]. It has also been demonstrated that post-meal hypertriglyceridemia has adverse effects on endothelial function [17, 18]. The exchange of core lipids between postprandial lipoproteins and low density lipoprotein and high density lipoprotein is increased during prolonged lipemia, resulting in the formation of highly atherogenic (small and dense) low density lipoprotein particles and reduced high density lipoprotein levels [19]. Therefore, a prolonged and high postprandial lipemia has the potential to increase the risk of developing cardiovascular disease [2, 15, 20] and other chronic diseases, especially in groups already at risk [21]. Figure 1 summarises the pathophysiological effects of postprandial hypertriglyceridemia.

After digestion, lipids present in food products are absorbed in the small intestine, packed into chylomicrons and transferred into the blood via the lymphatic system. The appearance of chylomicrons in the circulation is followed by an increase in liver-derived very low density lipoproteins (VLDL) due to competition for lipolysis between VLDL and chylomicrons [22, 23]. Thus, postprandial lipemia is a result of an increase in both intestine-derived chylomicrons and liver-derived VLDL. As chylomicrons are more readily targeted by lipoprotein lipase and the liver receptors, VLDL tend to increase in a greater extent than chylomicrons postprandially [23].

The rate at which lipids from individual foods and meals are digested, absorbed, incorporated into the blood stream and cleared depends on various non-modifiable factors (pathological conditions, genetic background, age, sex and menopausal status) as well as life style choices (physical activity, smoking, alcohol and medication use, dietary choices) [24]. The structure and the composition of the meal or food consumed are also an important factor in the control of postprandial lipemia, modulating the duration and the intensity of the postprandial response [25–27]. This article discusses the effects of those factors on the rate of appearance and clearance of lipids in the blood stream. We also make a case for blending the postprandial lipemic responses with the glycaemic response for the development of a novel tool for determining the healthiness of individual foods and mixed meals.

## Factors modulating postprandial lipemia

### Structure and composition of the meal or food consumed

The amount of dietary fat, as well as its fatty acid composition, has been demonstrated to influence postprandial triglyceride metabolism. Food structure, macronutrient and micronutrient composition have the potential to delay or expedite digestion and absorption of lipids; and therefore may also have an effect on the duration and intensity of the postprandial lipemia.

#### Lipid quantity

Postprandial triglyceride response to a meal has been shown to increase in proportion to the amount of fat in the meal in normal weight and obese individuals [28–31]. In normal weight and obese subjects an increase in the total fat content of a single meal increased postprandial chylomicron triglyceride response [30]. Postprandial investigation of obese boys has also demonstrated a greater increase in total plasma triglyceride levels after a high fat meal (about 68 g total fat) compared to a moderate fat meal (about 35 g total fat) [29].

#### Fatty acid composition and triglyceride structure

Evidence concerning the effect of fatty acid composition and triglyceride structure of the meal on postprandial lipemia is contradictory. It has been demonstrated that different dietary fatty acids modulate differently the plasma triglyceride peak concentration and the time of peak concentration as well as the rate of triglyceride clearance from plasma [32–43]. However, these studies are not consistent on their findings regarding the plasma triglyceride incremental area under the curve (iAUC). Some studies have reported no difference in plasma triglyceride iAUC between different fatty acids [36, 44], while other studies have reported lower plasma triglyceride iAUC after consumption of saturated fatty acid (SFA) rich meals compared with n-6 polyunsaturated fatty acids (n-6PUFA) and monounsaturated fatty acids (MUFA) rich meals [37, 39]. And another study has reported lower triglyceride iAUC after consumption of meals rich in n-6PUFA compared to MUFA and SFA [35].

The consumption of a dairy fat-based rich meal delayed plasma triglyceride peak time postprandially compared to a high n-6PUFA meal, although both meals yielded equivalent triglyceride iAUC and peak concentration over 8 h in overweight men [36]. Boham and colleagues [32] observed lower postprandial chylomicron triglycerides after the consumption of a dairy fat-based meal compared to a vegetable oil-based meal, despite observing no difference in total postprandial plasma triglycerides between test meals. Similar effect was observed in healthy young men consuming a saturated fat (dairy) rich meal compared with a n-6PUFA rich meal, with a more pronounced triglyceride peak in lipoproteins for subjects consuming a n-6PUFA rich meal [45].

Interventions comparing meals containing fatty acids in different positional configurations in the triglyceride have also presented conflicting results. Some studies have demonstrated a significant difference in the postprandial lipemia of subjects fed natural fats (palm oil and cocoa butter) compared to subjects fed interesterified fats [46–48]. However, other studies failed to demonstrate any difference in the lipemic response of subjects fed meals containing similar fatty acid composition with different positional configuration [49, 50].

Furthermore, as demonstrated by Weintraub and colleagues [51], postprandial lipemia is not only modulated by the fatty acid composition of the meal, but also by the fatty acid composition of a subject’s usual diet. Study subjects presented with a saturated fat challenge following chronic consumption of saturated fat experienced a more pronounced postprandial lipemia than subjects presented with an n-6PUFA challenge, following n-6PUFA chronic feeding or an omega-3 polyunsaturated fatty acid (n-3PUFA) challenge following n-3PUFA chronic feeding [51]. Chronic supplementation with long chain n-3PUFA has also been demonstrated to reduce postprandial lipemia in response to a fat challenge [52, 53].

#### Macronutrient composition

Some postprandial studies have demonstrated that the macronutrient composition of a meal has the potential to modulate postprandial lipemia. Different concentrations and type of carbohydrate consumed with a fat containing meal have been shown to change the postprandial triglyceride response to a meal. In a study with young males fed high fat meals, addition of glucose to the meal delayed triglyceride clearance [17]. It has also been demonstrated that glucose consumed with a high fat meal supresses postprandial triglyceride response in a dose dependent manner and that starch does not affect postprandial lipemia in young healthy subjects [54]. In contrast, a study in obese subjects consuming beverages containing various carbohydrate and protein concentrations, demonstrated an increase in postprandial plasma triglyceride iAUC with increasing carbohydrates and decreasing protein in the beverage [26]. Furthermore, normal weight and overweight subjects fed fatty meals, presented higher postprandial triglyceride response when the diet contained fructose compared to glucose [55].

Evidence suggests reduction in postprandial lipemia when a fatty meal is consumed with protein and that protein quantity and quality may also modulate postprandial lipemic responses [26, 56, 57]. Casein was found to cause a less pronounced postprandial lipemia (lower AUC) than whey protein in abdominally obese men when consumed as part of a high fat meal [58]. In contrast, in overweight and obese post-menopausal women, casein supported a larger triglyceride AUC than whey protein [56]. Additionally, whey protein led to lower postprandial lipemia when compared to cod fish protein and gluten in obese men and women [59]. In another study, fish protein did not affect postprandial lipemia compared to beef protein [40].

The fibre content of a meal has also been demonstrated to influence postprandial lipemia. Addition of partially hydrolysed guar gum to a high fat meal reduced the serum postprandial triglyceride iAUC in heathy subjects and tended to supress triglyceride peak concentration compared to a meal containing no fibre [60].

#### Food micronutrient composition

Polyphenols from berries have been shown to inhibit pancreatic lipase in vitro [61], thus potentially influencing postprandial lipemia. Indeed, strawberry polyphenol extract as part of a high fat meal lowered postprandial lipemia in hyperlipidemic subjects compared to a similar meal without polyphenols [62]. In contrast, meals containing 2 to 4 servings of blueberry or 400 g dealcoholized red wine as part of a fatty meal did not affect postprandial lipemia [63, 64]. Discrepancies may be due to different polyphenol concentrations in the test meals as well as differences in meal composition.

#### Food structure

Novel functional foods containing targeted dietary ingredients can be designed to reduce postprandial lipemia to minimise the risk of developing chronic diseases. The nature of the food matrix is known to influence the rate and extent of lipid release during digestion, therefore, can be expected to affect postprandial lipemia. Indeed, the increase in postprandial lipemia was much lower following consumption of a meal containing whole almond seed macroparticles, compared to almond oil mixed with defatted almond flour, suggesting that the cell wall encapsulating the almond lipids, plays an important role in determining the lipemic response [65]. Similar results have been observed in healthy male subjects fed either whole walnut or walnut oil [66].

In type-2 diabetic subjects, ingestion of isoenergetic meals including milk (liquid), butter (solid) or mozzarella cheese (semi-solid) showed a delay in the triglyceride peak after ingestion of the butter-based meal, possibly due to the presence of smaller fat globules in milk and cheese, which were digested at a faster rate than the fat in butter. The gastric emptying rate was greater with the cheese-based meal than with the milk-based meal [67]. In line with this study, healthy subjects have also demonstrated a delay in triglyceride peak after consumption of butter compared to milk [27]. Studies in rats showed that the ingestion of skim milk with added milk fat resulted in the faster appearance of plasma triglyceride and a sharper triglyceride peak than the ingestion of homogenized or non-homogenized cream [68]. Thus, the matrix structure and the oil − water interface have an impact on the physiological response after the ingestion of milk fat. In humans, daily consumption of butter led to higher fasting total and low density lipoprotein cholesterol than daily consumption of cheese [68]. In vitro studies have demonstrated that the size and interface composition of milk fat droplets modulate the rate of hydrolysis of the fat droplet by pancreatic lipase, playing an important role in digestion, absorption and consequently in the magnitude of postprandial lipemia [69, 70].

Furthermore, the use of different emulsifiers in food products as well as the size of the fat droplets has been shown to affect postprandial lipemia. In healthy males, oil finely emulsified in an oil-in-water system produced a faster more pronounced postprandial lipemia compared to a coarse oil-in-water emulsion [71]. Consumption of food emulsions containing different emulsifiers led to different postprandial triglyceride curves over 3 h; subjects consuming an emulsion containing Tween 80 presented higher postprandial lipemia than subjects consuming emulsions containing sodium caseinate and monoglyceride surfactant [25].

### Life style factors

#### Physical activity

The effect of physical activity on postprandial lipemia has been shown to vary with frequency, type and duration of exercise, and to be dependent on the composition of the meal consumed, energy consumed and the time of consumption [72]. Exercise prior to consumption of a fatty meal has been shown to increase postprandial triglyceride clearance and the degree of reduction appears to be linked with the energy expended [73–76] rather than the intensity of the exercise [72]. Data on the acute effect of exercise (up to 4 h prior to meal consumption) on postprandial lipemia is mixed. Some authors have demonstrated a reduction in postprandial triglyceride levels, while others have not observed a significant effect [72, 77, 78]. In contrast, exercise challenges performed 12 to 20 h prior to consuming a fatty meal consistently lower the postprandial triglyceride response.

It has also been demonstrated that postprandial lipemia increases with training cessation even for a period as short as 6 days; therefore, long term exercise training without recent training may not affect triglyceride metabolism and postprandial lipemia [79]. Indeed, lipoprotein lipase activity, suggested as the main enzyme responsible for the exercise-induced effects on postprandial lipemia, peaks between 4 to 18 h post exercise [72, 80, 81]. In addition, creating an energy deficit post exercise also seems to be important for reducing postprandial lipemia [72].

#### Smoking

Smokers have been shown to have a longer and more pronounced postprandial triglyceride response in plasma than non-smokers, due to a defective clearance of chylomicrons and chylomicron remnants [82, 83]. However, after smoking cessation, postprandial lipemia seems to decrease and the reduction is particularly significant for the lipoprotein fraction containing chylomicron remnants [84].

#### Lipid-lowering drugs

Pharmacologic reduction of plasma low density lipoprotein (LDL) cholesterol has been associated with an increase in the clearance rate of postprandial triglycerides in humans [85–88], suggesting that the triglyceride kinetics may be influenced by LDL cholesterol levels. In hyperlipidemic subjects, atorvastatin (statin) treatment has been demonstrated to improve triglyceride clearance in response to an oral fat challenge [86, 88] and chylomicron clearance in response to a chylomicron-like emulsion intravenous test [87]. Atorvastatin has also been shown to improve chylomicron metabolism by increasing chylomicron remnant catabolism in obese subjects [89]. Statins reduce de-novo synthesis of cholesterol by inhibiting the rate limiting enzymes, hydroxyl-methyl-glutaryl coenzyme A (HMG-CoA) reductase, consequently reducing the synthesis of VLDL and reducing circulating triglycerides to some extent [90]. In diabetic patients treatment with fibrates (gemfibrozil and ciprofibrate) has been shown to improve postprandial triglyceride levels [91, 92] and endothelial function [92]. In patients with metabolic syndrome, fibrates (Bezafibrate) improved remnant like lipoprotein clearance postprandially in addition to improving triglycerides and endothelial function [93]. Fibrates activate Peroxisome proliferator-activated receptor-α (PPAR-α) in the liver, increasing β-oxidation and lipoprotein lipase activity, and decreasing triglyceride secretion, consequently increasing clearance of VLDL and remnant lipoproteins [90]. Additionally, diabetic patients on a combined treatment with fenofibrate (fibrate) and simvastatin (statin) presented lower postprandial triglyceride iAUC compared with patients on a simvastatin only treatment [94].

Drugs used in the management of obesity may also contribute to the management of postprandial lipemia by inhibiting fat absorption, reducing overall food intake or improving fat distribution in viscerally obese subjects. Orlistat inhibits intestinal fat absorption by inhibiting intestinal lipases causing weight loss in obese individuals [95]. Sibutramine suppresses appetite and reduces caloric intake by acting centrally on neuronal receptors as an inhibitor of noradrenalin and serotonin, hormones involved in food intake [96]. Thiazolidinedione derivatives have also been used for the management of obesity [97] and Metformin has been used to improve insulin sensitivity, body weight, plasma lipids and leptin [98, 99].

#### Alcohol

Alcohol consumption has been shown to transiently enhance postprandial lipemia [63, 100] by acutely inhibiting lipoprotein lipase and causing a reduction in the breakdown of chylomicrons and VLDL remnants [101]. Its consumption has also been shown to increase hepatic synthesis of the large VLDL particles [102]. The acute effects of alcohol consumption on postprandial lipemia may be ameliorated by the regular practice of physical activity, but not by acute bouts of exercise. In a clinical study, physically inactive men had slower postprandial triglyceride clearance in response to a meal consumed with an alcoholic drink compared with habitual runners, who had their triglyceride clearance unchanged [103]. In contrast, acute exercise did not alleviate the effect of acute alcohol consumption on the postprandial lipemia of healthy moderately trained men and women [104].

Despite the acute effects of alcohol intake, case-controlled and epidemiological studies with diverse populations have established that a moderate intake of any alcoholic drink (wine, liquor, or beer) reduces the risk of cardiovascular disease [105–110]. This may be due to the fact that lipoprotein lipase activity seems to adapt during moderate (1–2 glasses) chronic alcohol intake [102].

### Biological factors

#### Nutrigenetics and nutrigenomics

Nutrigenetic and nutrigenomic studies have described the effect of genetic factors on post-prandial lipemia. Triglyceride metabolism is controlled by genes encoding the proteins involved in the synthesis of triglyceride-rich lipoproteins in the intestinal mucosa, their lipoprotein lipase mediated hydrolysis and the hepatic capture of chylomicron remnants via the interaction of the lipoprotein receptor with Apolipoprotein E and lipoprotein lipase (LPL). The available evidence links a number of candidate genes (APOA1/C3/A4/A5 cluster, ABCA1, CETP, GCKR, HL, IL-6, LPL, PLIN, and TCF7L2) to the modulation of postprandial triglyceride metabolism [111]. This, in part, explains the dramatic inter-individual variability observed in the postprandial lipemic response. A large majority of the published studies are limited to examining single-nucleotide polymorphisms (SNPs) of individual genes for their relation with specific traits. More recently efforts have been made to examine combinations of alleles that can provide better information about the architecture of the genes under consideration. This information is crucial and will pave the way for success of personalised nutrition for longevity and quality of life.

#### Gender

It has been demonstrated that male subjects have slower postprandial triglyceride incorporation into plasma and clearance compared to women [34, 112, 113] and that the magnitude of postprandial triglyceridemia is greater in men [114, 115]. Consistent with this concept, males have been shown to exhibit a greater plasma triglyceride response [113, 116], as well as increased postprandial free fatty acid levels [117], compared with female subjects. However, when the data were adjusted for visceral adipose tissue mass, the gender difference in postprandial plasma triglyceride response was eliminated, suggesting that the well-known gender difference in body fat distribution is also an important contributing factor. Men have a tendency to preserve excess fat in the abdominal (visceral) region, while women preferentially store fat in the subcutaneous areas of the buttocks and thighs [118]. The volume of abdominal fat, but not subcutaneous fat, has been inversely associated with suppression of fatty acid release from adipocytes, and free fatty acids are important sources of fatty acids for the assembly of VLDL [119]. Consequently, women have a more rapid clearance of fat resulting in lower postprandial triglyceride response compared to men [118].

#### Ageing

Postprandial lipemia has been shown to vary according to different age stages. In a clinical intervention, young subjects (20–30 years) had the fastest postprandial drop in triglyceride concentrations followed by middle aged subjects (31–40 years), while subjects aged 41–50 showed the longest elevation in triglyceride levels during the 6 h studied [120]. In other studies, the magnitude of the postprandial lipemia was greater in older compared to younger women [121] and triglyceride clearance was delayed in older compared to younger pre-menopausal women in response to an oral fat challenge [122]. In addition, the link between aging, postprandial lipemia and atherosclerosis has also been demonstrated in another study [123]. The mechanism behind this effect is uncertain. The reduction in the rate of gastric emptying, rather than intestinal motility, has been proposed to be responsible for exaggerated lipemia with increasing age. Since older individuals have a longer gastric emptying time, the absorption of fat can be expected to be slower, explaining the later increase in triglyceride levels. However, Krasinski et al. [124] have ruled out the possibility that the differences in lipemic behaviour observed in individuals under and above the age of 50 years are related to changes in the digestive absorptive processes, as the lipemic behaviour was similar with both intravenous infusion and oral ingestion of fat. Therefore, further investigation of the postprandial mechanism is needed. Nonetheless, the association of aging with postprandial lipemia may partly explain the influence of age on atherosclerosis.

#### Menopausal status

Postmenopausal women are known to have a more atherogenic lipid profile in general than pre-menopausal women, fact reflected in their postprandial lipemia. Post-menopausal women have presented higher postprandial triglyceride levels and delayed triglyceride clearance than pre-menopausal women in response to an oral fat challenge [122]. In other studies, post-menopausal women presented higher postprandial triglyceride levels compared to pre-menopausal women [125] as well as a delayed chylomicron response [126]. In contrast, Nabeno et al. [121] showed that the magnitude of the postprandial lipemia was not influenced by the menopausal status. The conflicting results observed among the interventions above may be due to differences in the fat load of the meal consumed.

##### Pathological conditions

###### Insulin resistance and diabetes

Insulin resistance increases circulating postprandial plasma triglycerides through a series of mechanisms. Insulin resistance in the adipose tissue stimulates increase in hormone sensitive lipase, increasing lipolysis and consequently, increasing the availability of non-esterified fatty acids (NEFA) in the circulation. NEFA are then up-taken by the liver and re-assembled in triglycerides, consequently driving an increase in the concentration and size of VLDL particles and an increased in the secretion of these particles. The excess NEFA also down regulates lipoprotein lipase (LPL) preventing the hydrolysis of triglycerides within the VLDL particle. The reduction in LPL activity also reduces the clearance of triglycerides from chylomicrons assembled after the consumption of a meal, impairing the clearance of chylomicrons and their remnant. In addition, in the insulin resistant state, secretion of Apolipoprotein B100 and Apolipoprotein B48 is increased [127].

Increased postprandial lipemia is an inherent feature of diabetic dyslipidaemia [128–130] in subjects with normal or elevated fasting plasma triglyceride levels. Type 2 diabetic males with prior myocardial infarction exhibited higher postprandial lipemic response than those without myocardial infarction, indicating that high responses may be a marker for a high-risk population [21]. An exaggeration of postprandial lipemia has also been reported in people with metabolic syndrome, a pre-disposition for the development of diabetes, compared to healthy subjects [131]. Microalbuminuria is a common feature in patients with type 2 diabetes mellitus and patients with this disease have been shown to exhibit higher postprandial triglyceride levels than those without microalbuminuria [132]. Furthermore, insulin therapy in diabetic patients has been shown to reduce the magnitude of postprandial lipemia after ingestion of a standard fatty meal [133].

###### Blood pressure

Hypertensive patients have been shown to have higher postprandial lipemia, compared to age and sex matched controls, following consumption of a fatty meal. Since hypertension is linked with insulin resistance, hyperinsulinemia in hypertensive patients may increase the hepatic production of VLDL, resulting in higher blood triglyceride levels following consumption of a fatty meal. Indeed data collected from the Framingham Heart Study demonstrated that postprandial triglyceride levels are inversely associated with high density lipoprotein cholesterol levels. Hypertensive males have presented higher postprandial triglyceridemia and delayed triglyceride clearance compared to healthy males in response to an oral fat challenge [131, 134]. A link between hypertension, postprandial lipemia and atherosclerosis has also been demonstrated in another study [123].

###### Obesity

Obese subjects have been demonstrated to present with higher postprandial triglyceridemia and slower triglyceride clearance from plasma then healthy normal weight subjects [30, 135, 136]. Although obese subjects may present normal fasting lipemia, their lipid metabolism is in general abnormal and, postprandially, may lead to increased triglyceride rich lipoproteins in circulation. Fat accumulation in the abdominal region seems to be associated with increased postprandial lipemia in men and women [137–139]. After an oral fat challenge, postprandial triglyceride levels were elevated in obese compared to normal weight women [137, 138], and abdominally obese women (waist to hip ratio > 0.80) presented higher postprandial triglyceridemia than other obese women [138]. Viscerally obese men had a slower chylomicron clearance compared to normal weight men. The slower clearance rate of chylomicrons and plasma triglycerides in these subjects may be either due to a reduction in low density lipoprotein receptor expression or due to excess VLDL triglyceride, which may have an increased secretion rate or a decreased clearance rate [139, 140]. However, other mechanisms may also be involved.

## Discrepancies among previous postprandial studies

Although a plethora of studies is available on fat challenges and the postprandial effects of different meals, the lack of standardization among those studies prevents an accurate comparison and estimation of the effects of single foods and specific fatty acids on postprandial lipemia and generates discrepancies. Studies on fat or meal challenges differ in a number of parameters. They have assessed meals containing a wide range of fat contents, from 10 g to over a 100 g of fat in a single feed. They have analysed triglycerides or retinyl-palmitate in a variety of sample types, including whole blood, plasma, serum, chylomicrons and remnant lipoprotein, over a wide time range from 2 to up to 12 h postprandially. Delivery methods and target population are also variable. Often an unequal number of males and females, as well as subjects within a broad age range, have been recruited. In addition, in most of these studies, subjects with a pathological condition have been recruited instead of healthy normal weight subjects, incorporating extra variables to an already complex equation. Therefore, there is a need for the standardization of postprandial studies to improve the comparison of the effects of different food products on postprandial lipemia.

## Future directions

Subjects with metabolic syndrome, obesity and hypertension, among other disorders, may have normal fasting blood lipids, despite presenting with elevated postprandial lipemia. Considering that individuals are in the postprandial state for most of the day, a more effective measure of abnormal lipemia needs to be developed. Furthermore, a subject, independent of his/her health status, should be able to choose meals that promote lower lipemic responses postprandially, in order to reduce postprandial inflammation and the risk of developing or aggravating chronic disease.

Currently, the glycaemic index and the glycaemic load are used to grade foods and meals as a determinant of their healthiness. However, the glycaemic index can be altered by addition of fat [141]. As a result a fat enriched food produces a low glycaemic index, but may not necessarily be overall healthy, as it can increase postprandial lipemia and consequently increase inflammatory response [142, 143].

Ooi and colleagues [144] have suggested the development of an index to measure the effect of different meals on postprandial lipemia. They proposed this index should be measured as the triglyceride’s iAUC in response to a test meal divided by the triglyceride’s iAUC in response to the standard meal multiplied by 100. Unlike the glycaemic index, the lipemic index would potentially be greater than 100% for some food products. However, it can be argued that considering only the triglyceride’s iAUC may be a very simplistic way of measuring the effect of different food products on postprandial lipemia. As discussed in this article, despite not resulting in different postprandial triglyceride iAUC, different foods may modulate triglyceride peak time and magnitude differently; a fact that is masked by adopting a single measure based on the iAUC. The term lipemic index may also be misleading as it is used in the clinical setting to define the quality of the plasma or serum sample for analysis, being used as a synonym of turbidity [145].

We propose the development of a new tool to aid the selection of food products based on a smaller and steady postprandial rise not only in blood glucose but also in blood triglyceride levels. Foods with low glycaemic and lipemic responses have the potential to improve satiety and consequently reduce caloric intake for the prevention of obesity and related cardio-metabolic diseases. Developing a ranking criterion based on both glucose and lipid responses may help consumers make healthier choices and avoid health complications.

## Conclusions

Postprandial lipemia, characterized by a rise in circulating triglyceride containing lipoproteins after the consumption of a meal, is a dynamic, non-steady state condition to which humans are exposed for most of their day. Evidence accumulated over the years demonstrates that postprandial lipemia may modulate endothelial function and homeostatic variables, including blood coagulation factors, platelet function and pro-inflammatory cytokine expression. Therefore, suggesting that postprandial lipemia should be included in the assessment and treatment of cardiovascular risk factors.

As discussed in this review, food structure and composition are important determinants of postprandial lipemia and merit further examination to delineate the role of different natural and processed foods to human health and disease. Foods and meals that improve postprandial triglyceride concentrations are likely to play a vital role in healthy human diets, warranting the need for the development of a standard methodology to determine the extent and duration of postprandial lipemia. In addition, the glucose metabolism is of equal importance for the healthiness of the foods we consume and should be considered in conjunction with the lipid metabolism in the development of a novel index to determine the healthiness of the foods.

## Abbreviations

AUC: Area under the curve
HMG-CoA: Hydroxyl-methyl-glutayl coenzyme A
IAUC: Incremental area under the curve
ICAM-1: Intercellular Adhesion Molecule 1
IL-8: Interleukin-8
LDL: Low density lipoprotein
LPL: Lipoprotein lipase
MUFA: Monounsaturated fatty acids
n-6PUFA: n-6 polyunsaturated fatty acids
NEFA: Non-esterified fatty acids
NF-κB: Nuclear factor κB
ROS: Reactive oxygen species
SFA: Saturated fatty acid
SNPs: Single-nucleotide polymorphisms
TLR4: Toll like receptor 4
TNF-α: Tumour necrosis α
VLDL: Very low density lipoproteins
